# The SOFIA pilot trial: a cluster-randomized trial of coordinated, co-produced care to reduce mortality and improve quality of life in people with severe mental illness in the general practice setting

**DOI:** 10.1186/s40814-021-00906-z

**Published:** 2021-09-03

**Authors:** M. P. Rozing, A. Jønsson, R. Køster-Rasmussen, T. D. Due, J. Brodersen, K. H. Bissenbakker, V. Siersma, S. W. Mercer, A. D. Guassora, J. Kjellberg, P. K. Kjellberg, M. H. Nielsen, I. Christensen, J. E. Bardram, F. Martiny, A. Møller, S. Reventlow, on behalf of the SOFIA Study Group

**Affiliations:** 1 The Section of General Practice and the Research Unit for General Practice, Department of Public Health, University of Copenhagen, Copenhagen, Denmark; 2 Psychiatric Centre Copenhagen, Outpatient clinic for geriatric psychiatry, Copenhagen, Denmark; 3 The Primary Health Care Research Unit, Region Zealand, Denmark; 4 Old Medical School, University of Edinburgh, Edinburgh, UK; 5 VIVE - The Danish Center for Social Science Research, Copenhagen, Denmark; 6 Copenhagen Center for Health Technology (CACHET), Department of Health Technology, Technical University of Denmark, Lyngby, Denmark

**Keywords:** Pilot study, Severe mental illness, Mortality, Morbidity, Quality of life, Primary care

## Abstract

**Background:**

People with severe mental illness (SMI) have an increased risk of premature mortality, predominantly due to somatic health conditions. Evidence indicates that primary and tertiary prevention and improved treatment of somatic conditions in patients with SMI could reduce this excess mortality. This paper reports a protocol designed to evaluate the feasibility of a coordinated co-produced care program (SOFIA model, a Danish acronym for Severe Mental Illness and Physical Health in General Practice) in the general practice setting to reduce mortality and improve quality of life in patients with severe mental illness.

**Methods:**

The SOFIA pilot trial is designed as a cluster randomized controlled trial targeting general practices in two regions in Denmark. We aim to include 12 practices, each of which is instructed to recruit up to 15 community-dwelling patients aged 18 and older with SMI. Practices will be randomized by a computer in a ratio of 2:1 to deliver a coordinated care program or usual care during a 6-month study period. A randomized algorithm is used to perform randomization. The coordinated care program includes educational training of general practitioners and their clinical staff educational training of general practitioners and their clinical staff, which covers clinical and diagnostic management and focus on patient-centered care of this patient group, after which general practitioners will provide a prolonged consultation focusing on individual needs and preferences of the patient with SMI and a follow-up plan if indicated. The outcomes will be parameters of the feasibility of the intervention and trial methods and will be assessed quantitatively and qualitatively. Assessments of the outcome parameters will be administered at baseline, throughout, and at end of the study period.

**Discussion:**

If necessary the intervention will be revised based on results from this study. If delivery of the intervention, either in its current form or after revision, is considered feasible, a future, definitive trial to determine the effectiveness of the intervention in reducing mortality and improving quality of life in patients with SMI can take place. Successful implementation of the intervention would imply preliminary promise for addressing health inequities in patients with SMI.

**Trial registration:**

The trial was registered in Clinical Trials as of November 5, 2020, with registration number NCT04618250.

Protocol version: January 22, 2021; original version

## Background and rationale

People with a severe mental illness (SMI) on average die younger than the general population. It is projected that among people with SMI, defined as psychotic conditions, bipolar disorder, and severe depression [1], between 10 to 20 life years are lost [2]. Unnatural causes of death, i.e., suicide, accidents, and homicide, considerably contribute to this disparity [3], yet chronic somatic health conditions account for the majority of lost life years among people with SMI [4]. The majority of deaths are due to cardiovascular disease, respiratory disease, and cancer [5–9]. People with SMI are at higher risk of several chronic somatic conditions, particularly cardiovascular diseases [10], diabetes mellitus type II [11], metabolic syndrome [12], respiratory diseases, and liver abnormalities [13] as well as the risk of somatic multimorbidity [9]. The mechanisms connecting SMI and somatic conditions are complex. Contributing to the higher risk of somatic conditions in SMI patients are unhealthy living conditions, the adverse metabolic effects of long-term psychotropic medication use, and possibly shared genetic and metabolic vulnerability [14, 15]. Finally, lower adherence to treatment regimens and poorer medical treatment are at play. Preliminary evidence suggests that the mortality gap for people with SMI is amenable to intervention and that primary care may play a pivotal role in reducing this gap [16].

People with SMI generally have a lower self-reported quality of life and have a lower socioeconomic status. Likewise, the label “mental illness has proven to have a severe negative social impact after being diagnosed, since all further actions and sentiments are likely to be understood by others in relation to, and as confirmation of, the patient’s status as mentally ill” [17–19].

In the SOFIA study, an intervention was developed by combining state-of-the-art evidence-based clinical, and social knowledge, with the perspectives of all involved parties in a participatory co-design process [20]. Furthermore, barriers for trans-sectoral treatment of patients with SMI, and potential solutions for these, were explored in a series of workshops, focus groups, and interviews as part of the co-design phase to help guide the development of an intervention [21]. This intervention essentially aims to improve the treatment of comorbid somatic conditions in persons with SMI through an enhanced educational training of general practitioners and their clinical staff, and by implementing strategies to better accommodate patients with a psychiatric background in primary care. It is hypothesized that a focus on the individual patient’s needs and preferences will enhance the relationship between general practitioners and patients with SMI and thereby improve quality of life. Practically, this entails an active outreach to patients with SMI in the general practice setting, longer and holistic [22] consultations with attention paid to both quality of life and disease management, use of a conversation tool, an individualized care plan, and balancing treatment options against the patient’s needs, abilities, and individual circumstances. We hypothesize that the enhanced relationship will encourage patients with SMI in the general practice setting to attend health consultations systematically and regularly, which in combination with patient-centered care will improve timely detection and treatment of somatic morbidity. Ultimately, following this, we hypothesize that this intervention will reduce excess mortality and enhance the needs-based quality of life in patients with SMI.

In short, the intervention includes prolonged clinical consultations with full reimbursement, a full-day introductory educational course for general practitioners and practice staff, and the distribution of a handbook promoting trans-sectional care. Components of the design and intervention, similar to those from the SOFIA intervention, have been used and assessed in previous trials [23, 24]. However, none of these interventions were specifically aimed at patients with severe psychiatric morbidity in the general practice setting. Furthermore, few studies have looked into how general practice can improve the lives of people with multimorbidity or have investigated the efficacy of training doctors to manage people with multimorbidity [25]. Therefore, in light of this paucity of evidence regarding management of care in this patient group in the general practice setting, conducting an RCT raises significant practical concerns. Moreover, when developing a complex intervention, a scaled approach is recommended, i.e., from small-scale feasibility studies to larger-scale pilot trials before an RCT can take place to ensure that the intervention is sequentially optimized and is implementable, cost-effective, and acceptable to patients and providers [26].

Before this pilot study, we conducted a small-scale single-arm feasibility study, which demonstrated that the recruitment to and completion of the prolonged consultation was feasible in general practice (unpublished raw data). However, we identified several challenges regarding the optimal method for recruitment of patients, ensuring implementation of the intervention in practice, optimizing the data collection process, and the quality of data. Also, the educational component of the intervention was not tested in the small-scale single-arm feasibility study, and neither was the impact of randomization on patients’ willingness to participate and on practices’ behavior. Therefore, we will undertake a cluster randomized pilot trial before assessing the efficacy of the SOFIA intervention in a larger-scale RCT. We considered usual care as an appropriate comparator as it likely approaches the naturalistic conditions for patients with SMI in the general practice setting.

The primary objectives of this pilot study are:

1. To assess the implementability of the intervention including the fidelity and acceptability of the intervention for patients and general practitioners
2. To assess the feasibility of the design in terms of recruitment of practices and patients and to assess retention of patients and practices during the intervention
3. To assess the feasibility of collection of outcome measures and thereby obtain preliminary data to inform the required sample size in a definitive, large-scale efficacy trial—hereafter referred to as ”The SOFIA Trial”

The secondary objectives of the study are:

1. To examine whether our recruitment strategy is at risk for preferential inclusion of patients with a favorable prognosis
2. To examine to which extent general practitioners’ use of patients’ response to the MultiMorbidity Questionnaire (MMQ) (see section “Data collection before randomization” for a description of the MMQ below), measuring quality of life in patients with multimorbidity, during the prolonged consultations threatens the validity of the questionnaire as an outcome measure
3. To assess and inform selected aspects of the program theory relating to the prolonged consultations and the educational course

## Methods

### Trial design

The pilot study is designed as a randomized, non-blinded, parallel-group trial. A 2:1 cluster randomization will be performed with the general practice level as the unit of randomization.

### Trial setting

The study will be conducted in the Capital Region of Denmark (excluding the island Bornholm) and the Region of Zealand. Region Zealand includes the island Zealand, which it shares with the Capital Region, and the adjacent islands, Lolland, Falster, and Møn. In these two regions, approximately 1600–1800 patients are enlisted per general practitioner.

### Eligibility criteria

#### In- and exclusion criteria of general practices

General practices have to be located in the Capital Region of Denmark and the Region of Zealand. General practices without permanent staff, so-called regional clinics or clinics run by private firms, are not included due to lack of continuity of care, which is assumed to be a critical underlying component of the hypothesized effect of the intervention. General practices that have participated recently or still participate in trials similar to the SOFIA trial will be excluded. No other in- or exclusion criteria will be applied when recruiting general practices.

#### Recruitment of general practices

General practices will be invited to participate by the research team. More than one general practitioner may be employed at a single practice. To participate in the trial, all general practitioners employed at a practice should agree to participate. We aim to recruit 6 general practices in the Capital Region and 6 general practices in Region Zealand. Each Region is divided into multiple municipalities. We aim to recruit practices from different municipalities to increase the generalizability of the pilot study to inform the feasibility of a larger-scale RCT. Practices are recruited via telephone, followed by an email with information about the study and a further telephone call in which practices confirm or decline participation. General practices confirming interest in participating in the study will be visited by members of the research team. During this visit, practices will be provided instructions and materials for recruitment of patients, data collection, and any other information needed to conduct the trial.

#### Inclusion criteria primary care patients with SMI

Patients will be included if they:

- A. Are aged 18 and over on the day of inclusion of the general practice

*And*

- B1. Have a psychotic disorder

[Registered at general practice with International Classification of Primary Care version 2 (ICPC-2) psychiatric diagnosis p72 (psychotic disorders) which roughly corresponds to International Classification of Diseases 10 (ICD-10) codes F20-F29.9]

*Or*

- B2. Have a bipolar disorder

[Registered at general practice with ICPC-2 diagnosis of p73, bipolar mood disorders, roughly corresponding to ICD-10 codes F30-F31, and F34.0; *Or* have a prescription of lithium (Anatomical Therapeutic Chemical classification (ATC) N05AN); *Or* are registered at general practice with ICPC-2 diagnosis p76, unipolar depressive disorders, roughly corresponding to ICD-10 codes F32.2 - F32.3, and/or F33.2- F33.3, and F53.0; *And* have a prescription of Lamotrigine (N03AX09) and/or Carbamazepine (N03AF01) and/or Valproic acid (N03AG01)]

*Or*

- B3. Have a severe depressive disorder.

[Registered at general practice with ICPC-2 diagnosis p76, unipolar depressive disorders roughly corresponding to ICD-10 codes F32.2 - F32.3, and/or F33.2- F33.3, and F53.0; *And* have a prescription of tricyclic antidepressants (ATC: N06AA) and/or Selective Norepinephrine Receptor Inhibitors (SNRI) venlafaxine (N06AX16) and duloxetine (N06AX21) and/or Monoamine Oxidase A Inhibitor (MAOI) (N06AG) and/or non-selective MAOI (N06AF).]

#### Exclusion criteria primary care patients with SMI

Patients will be excluded from participation if they are:

A. Subjected to any type of legal measure as stipulated in the Danish Mental Health Care Act (Psykiatriloven), e.g., forced detention or medication;
B. Registered with a dementia diagnosis ICPC-2 p70, roughly corresponding to ICD-10 F0.01-0.03 or registered with organic psychosyndrome or other neurological diseases (ICPC-2 P71, N73, N99)
C. Receiving end-of-life care
D. Non-Danish speakers
E. Psychiatric diagnosis appears to be incorrect or outdated
F. Assumed by the patient’s general practitioner to have an overall functional level that is too low for meaningful participation in the trial (use of this criterion will be evaluated on an individual basis by the trial management team to minimize the risk of selective inclusion).

#### Identification, eligibility assessment, and recruitment of patients with SMI

Participating practices will be instructed to collect the International Classification of Primary Care version 2 (ICPC-2) codes and ATC-codes from the electronic medical record on all patients who are registered at the practice at the commencement of the study and send these to the research team. ICPC-2 codes are routinely used in Danish medical practice.

A computer algorithm will randomly select a sample of 45 patients who fulfill the externally verifiable inclusion criteria for each participating general practice. This selection will be stratified, in that each sample of 45 patients includes 15 patients who meet diagnostic criteria B1, B2, or B3 respectively. The research team sends this list of 45 potentially eligible patients to the general practitioners. General practitioners are instructed to screen these lists for correct diagnoses. In case of uncertainty regarding diagnosis, general practitioners should contact the research team. When a diagnosis is verified by the general practitioner, the general practitioner will assess the eligibility of the listed patients according to the exclusion criteria described above. General practitioners are instructed to document any reasons for exclusions and any reasons for deviating from the exclusion criteria. General practitioners are instructed to recruit at least 2 patients from each diagnostic subgroup (corresponding to the inclusion criteria B1, B2, and B3). We employ a reimbursement limit of 15 patients per practice. Patients participating in the study will not receive financial compensation.

The general practices will establish the initial contact with patients who were found eligible. Practice staff will contact potentially eligible patients via telephone, and provide brief oral information about the study. When approaching patients, practice personnel are instructed to register the date and means of contact, e.g., telephone or email, and the number of attempts. In case the patient does not respond, an email, text message, or message on voicemail is sent. If a patient cannot be reached after at least three attempts, the patient will be excluded from participation. Patients, who express interest in participating in the study, will be invited to an initial 10–30-min enrolment visit at the general practice. General practices are instructed by the research team to send an information leaflet and a copy of the informed consent form by mail or email to patients before they visit their practice. During the enrolment visit, further questions regarding the study will be discussed. If patients agree to participate, written informed consent will be obtained. Moreover, all patients receive the questionnaires MMQ and the EQ-5D-5L measuring quality of life and health status after they visited their practice. Patients can either choose to receive the questionnaire in print from their general practice or via their e-Boks (provider of secure platforms and digital post-boxes) from the research team. A more detailed description of the properties of these questionnaires is provided below in the section “Data collection before randomization”—quality of life measurements. Patients are asked by the general practitioner to complete these questionnaires online through a link in their secured email or return them by mail in a prepaid envelop to the research team.

### Interventions

#### Intervention group

##### Prolonged clinical consultations: the SOFIA consultation

Patients, whose general practice has been allocated to the intervention arm, will be invited to a prolonged consultation after the general practitioners have attended an introductory educational course (see below). All patients will be encouraged to bring a relative or social worker if applicable. Non-attenders will be contacted to reschedule missed appointments.

The prolonged consultation will be held by the general practitioner and will follow the SOFIA scheme, which is a further development of the existing consultation practice and communication skills as taught during Danish general practitioners’ specialty training [27]. In the consultation, SOFIA stands for SOcial, FInding, and Agreement, which guides the flow of the SOFIA consultation. The structure of the prolonged consultation is described in Table 1. The consultation will primarily focus on somatic health problems. The consultations will last up to 45 min, which includes time for preparation, ordering paraclinical testing, and referrals. Paraclinical tests and other indicated point-of-care tests will be performed by practice staff.

**Table 1:** The SOFIA scheme

**Welcome** Patient and general practitioner agree on the aim of consultation. Information about the study and participation is repeated. It is orally confirmed that informed consent for study participation has been given.	
***SOcial clinical space: The “patient part” of the consultation*** This opening part of the consultation aims to establish a positive relationship between the patient and the general practitioner. The patient has the opportunity to present his or her complaints and through clarifying the patient’s thoughts, feelings, and notions regarding these complaints. The general practitioner sets an agenda for the consultations. Suggestions for open questions the general practitioner could ask are: “How are you? Is there anything that you would like to focus on today? Are there any other concerns that I should be aware of? Is there anything in particular that you hope to gain from today’s meeting and is there anything that you hope that I can help you with?” Dependent on the study arm the patient is allocated to, results from MMQ1 may be discussed. The general practitioner is instructed to probe for areas that need attention and needs that should be focused on, especially if the patient’s sum score in any of the six scales indicates poor quality of life in the construct measured by the scale. The general practitioners are instructed to ask, whether the patient experiences suicidal thoughts (if so general practitioners are instructed to follow the SOFIA handbooks’ guide on talking about suicide). If not already known, general practitioners ask about possible substance abuse, self-harm (if yes, see the SOFIA handbook for referrals).	
***FI*nd any symptoms for undiagnosed or undertreated somatic diseases: The “general practitioners’ part” of the consultation** The middle section of the consultation aims to collect information on current diagnoses and their treatments and to detect possible, unrecognized, and undertreated disorders or overdiagnosed and/or overtreated conditions. The general practitioners are instructed to ask about known diseases and current treatments and any symptoms that the patient may experience. The general practitioners will perform a focused physical somatic diagnostic interview, based on any somatic concerns that the patient and general practitioner agree upon. The patient must be physically examined, even if the patients have no physical complaints, because of the delayed and altered bodily experience often accompanying SMI. The general practitioners conclude this part of the consultation with a brief review of current medication and, if relevant, make a plan to optimize pharmacological treatment. The general practitioners discuss adherence challenges related to treatment, possible side effects, and any possible considerations or wishes for medication changes with the patient. If required, a pharmacologist can be consulted by email. If required, a follow-up consultation focusing on medications will be scheduled.	
***A*gree on individual care plan (final step of the SOFIA consultation)** During the final part of the consultations, an individual care plan is made. The general practitioner and the patient will discuss current treatment with the patient, i.e., is the patient adequately treated for his/her current conditions. The general practitioner and patient assess whether treatment adjustments are needed. The general practitioner explores if anything discussed during the consultation requires follow-up, i.e., referrals to municipality or psychologist, or referral to “institutional care facility” or other services as provided by the SOFIA handbook. The general practitioner creates a safety-net—by emphasizing that the patient is always welcome to contact the practice. If medically indicated, paraclinical tests and follow-up consultations will be scheduled.	

##### Introductory educational course

A mandatory 1-day course for all general practices assigned to the intervention group will be held. The course aims to provide additional education on challenges and pitfalls when delivering care for patients with SMI and to instruct general practitioners on how to conduct the SOFIA consultation. An academic general practitioner will provide hands-on tools for the clinical and diagnostic management of this patient group. Then, a workshop will be conducted on how to include patients with SMI’s individual social and cultural aspects during the consultation and in the planning of treatments, including how to tackle, i.e., substance abuse. The training will also include how to do a medication review, taught by a pharmacologist, and practical information about participating in the study. Finally, a patient will be providing the patient perspective of being treated for somatic diseases while also suffering from SMI. This will add to the training’s final hands-on sessions on conducting the SOFIA consultation. The training is built upon the pedagogical principles of transfer [28] and will be followed up by a pre-organized peer-to-peer session and continuous support from the project office. The training will be assessed in a feasibility study in which fidelity and acceptability will provide a deeper insight into how the training is transferred and maintained in a clinical everyday setting, rather than solely evaluating the participants’ satisfaction with the course.

##### Promoting trans-sectional care—the SOFIA handbook

Owing to the Danish organization of health care and social care into different sectors resorting under separate laws, formal trans-sectional care plans are not feasible. However, each general practitioner assigned to the intervention arm will receive a specialized handbook, specific for their municipality and regional affiliation, containing contact information of relevant social or health care actors, and helplines in case of substance abuse and an increased risk of suicide and self-harm.

##### MMQ part 1

Apart from improving physical health, the SOFIA study aims to improve the quality of life among the participants. To measure the quality of life, we use the patient-related outcome measure, MMQ. MMQ was developed for patients with multimorbidity, including SMI. In the SOFIA pilot trial, we want to examine if the simultaneous use of MMQ as a conversation tool and an outcome assessment will bias the MMQ as an outcome measure. Therefore, the general practices will be cluster-randomized in two subgroups, with 2 general practices from each region in both subgroups. All patients are asked to complete the MMQ part 1 (MMQ1) before the prolonged consultation. In the first subgroup, the relative sum-scores of six scales will be provided to the general practitioners via an electronic link. During the educational course, the general practitioners in the first subgroup are instructed on how to use the six scales’ sum-scores as a clinical conversation aid during the “patient part” of the consultation. In the other subgroup, the general practitioners will not have access to the MMQ1 scores.

#### Control group

Primary care patients in the control group will receive usual care during the study period. In the control group, the general practitioners will not have access to the MMQ1 six scales’ sum scores. Usual care includes free access to primary healthcare both during and out of office hours. Of relevance in this context is that in Denmark all primary care patients suffering from chronic conditions, i.e., diabetes, cardiovascular diseases, COPD and asthma, rheumatoid arthritis, osteoporosis, thyroid conditions, and all mental conditions, are offered an annual disorder-specific health-check, and medication and treatment review by their general practitioner.

#### End-of study

The study period ends on September 30, 2021. All patients in the intervention and control groups receive the same questionnaires as at baseline (MMQ, EQ-5D-5L) to be returned to the research team by mail or through RedCap. This concludes the study period for all participating patients.

### Concomitant care

Any new interventions or initiatives introduced by local health authorities, municipalities, researchers, health organizations, and the like, aimed at improving healthcare for persons with psychiatric conditions, will be recorded during the study.

### Outcomes measures

The overall aim of the pilot trial is to assess the feasibility and quality of trial management, intervention content, and implementation. To qualify the content of the intervention and its implementation, a formative process evaluation of the pilot trial will be conducted with a twofold purpose: to assess the implementation platform and to inform the definitive SOFIA trials’ preliminary program theory [29]. The formative aspect of the evaluation will ensure more rapid feedback which can give rise to iterative changes during the pilot trial after which the results of these adjustments can be assessed. This formative approach will maximize the potential for an optimum model before the definitive trial [29]. The study design is a mixed-method study using data from an electronic data registration system (REDCap, see below), registers, and qualitative methods (interviews and observations). For the objectives, where threshold criteria are given, these will be used to decide if, and in that case, how to progress to the large-scale RCT. Not reaching these thresholds does not necessarily indicate unfeasibility but will be an incentive to re-evaluate and possibly adjust the design and/or elements of the intervention.

#### Primary outcome measures

1. To assess the implementability of the intervention

Concerning the implementation, we will assess the implementation platform for the intervention by assessing fidelity (if and to what extent the intervention was performed as planned and the educational recommendations were followed) and barriers and facilitators for implementation concerning, e.g., comprehensibility, acceptability by practices, preparation, and the integration of the intervention into existing practices. This will be done by performing qualitative semi-structured interviews with general practitioners and staff in the intervention group during the pilot trial and observations of the introductory course and prolonged consultations.

2. To assess the feasibility of the design in terms of recruitment of practices and patients and assess retention of patients during the intervention

The feasibility of the recruitment of practices and patients will be assessed using the proportion of contacted general practices, which agree to participate in the study and the eligibility and consent rate of patients. We consider a participation rate of at least 20% of the contacted general practices and at least 60% of the contacted patients as an indication that our recruitment strategy is feasible. Based on registrations by the practices in REDCap, feedback and questions regarding support from practices during the process, and interviews with general practitioners and staff after the recruitment process, we will assess the appropriateness of the recruitment process and the introduction and support to the participating practices.

Retention of patients will be assessed as the proportion of recruited patients in the intervention group who attend the first consultation, and the minimum threshold for attendance is set at 90%. Information on measures such as non-return of consent forms, non-shows by participating patients in general practice, and difficulties reaching patients will be collected. Based on interviews with the practices, we will explore their work related to ensuring patient attendance.

3. To assess the feasibility of collection of outcome measures and thereby obtain preliminary data to inform the required sample size in the definitive trial

All patients, regardless of allocation, will complete the two outcome questionnaires the MMQ and the EQ-5D-5L questionnaire at baseline, after randomization, and at the end of the pilot trial. Based on the number of returned questionnaires, we will calculate response rates and the proportion of missing data in each completed questionnaire. We set a minimum acceptable threshold for the response rate before randomization of both questionnaires at >90% with a maximum proportion of missing data at <10%. We set a minimally acceptable response rate at >90% for both questionnaires at the end of the study period in both the intervention and the control group.

Data on mortality and in- and outpatient admissions during the study period in the participating patients will be collected from the national patient registries after completion of the pilot trial. These data, along with the scores of the MMQ, and the EQ-5D-5L questionnaires, will be used to estimate the necessary sample size to power the definitive trial, which primary endpoints are mortality and quality of life, as measured by the MMQ.

#### Secondary outcome measures

1. To examine whether our recruitment strategy is at risk for preferential inclusion of patients with a favorable prognosis

To assess if, and to what extent, selective recruitment is present in our study, we aim to use national patient registries and registrations in REDCap to examine potential differences in demographic, socio-economic, and health-related characteristics between eligible patients who agreed and who declined participation in our study.

2. To examine to which extent use of the quality of life questionnaire (MMQ1) during the prolonged consultations threatens the validity of the questionnaire as an outcome measure

It is the intention, in the definitive trial, that the general practitioners can use the MMQ1 as a conversation tool to help them identify issues that should be addressed during the consultation. In this scenario, in the intervention arm, the general practitioners have access to the aggregated patients’ MMQ scores, and patients might have a different understanding of the questions after having discussed them during the consultation as opposed to the participants in the control group. This might compromise the validity of the MMQ1 as a primary outcome in the definitive trial. Within the pilot study, we aim to empirically test for this risk of bias. To this end, in randomly selected 50% of the intervention practices, the general practitioner will have access to the aggregated score of selected domains in the MMQ1. In the remaining 50% of the intervention practices, MMQ1 scores will be concealed for the general practitioner. This sub-study is designed as a small equivalence trial nested in the pilot study. The aim is to show that the two approaches are not different in terms of mean change in MMQ1 scores before and after the intervention by using the scores of MMQ2 and EQ-5D-5L as anchors.

3. To assess and inform selected aspects of program theory relating to the prolonged consultations and the educational course

Concerning the program theory, we will assess selected proposed connections, mechanisms, and contextual conditions influencing intervention implementation and outcomes, mainly to gain a preliminary indication of the effectiveness of the prolonged consultations (change in diagnosis, medication, referrals, increased contact) and the effectiveness of the educational course in changing the clinical practice approach. This can give rise to elaborations of the program theory and adjustments of the intervention before the definitive trial. These issues will be assessed by a combination of the registrations in REDCap, observations, and interviews with practices in the intervention group. Furthermore, we will conduct interviews in all allocation groups on usual care and in the control group on the non-study care they have received during the study period.

### Participants’ timeline

An overview of the schedule of enrolment, interventions, assessments, and visits for participants is given in Table 2.

**Table 2:** Participants’ timeline

	Study period			
	Enrolment	Allocation	Post-allocation	Close-out
Timepoint	*− 5 months*	*0*	*>0–6 months*	*>6 months*
Enrolment:				
Recruitment general practices	X			
Identification patients	X			
Eligibility screen patients	X			
Informed consent	X			
Allocation		X		
Interventions:				
Educational course general practitioners			X	
Prolonged consultations			X	
Assessments:				
Patient demographics	X			
MMQ1	X	X		X
MMQ2	X	X		X
EQ5D-5L	X			X
Data on retention patients				X
Mortality data				X
Socioeconomic status				X
Paraclinical test data				X
Medication use				X
Hospital admission data				X
Ethnographic Interviews	X	X	X	X

### Power analysis and sample size

As this is a pilot trial, a formal sample size calculation is not performed. We aim to include 12 practices, each recruiting up to 15 patients per practice, amounting up to 180 patients in total. We considered this number sufficiently informative on the practicalities of delivering the intervention and collecting outcome data.

### Allocation and concealment

Unit of randomization will be the general practice so that all participating patients within the general practice receive either the intervention or care-as-usual. General practices will be randomly assigned on a 2:1 basis to either the intervention or care-as-usual group in blocks of three within each region. A block of three practices will be allocated simultaneously after completion of the baseline documentation for all study participants within the practices. Within each randomization block of 3 practices, we further randomize 1 of the 2 practices in the intervention arm to a sub-group, where the general practitioner has access to the aggregated scores of the MMQ1. The other intervention practice within the randomization block of 3, will be allocated to a sub-group in which MMQ1 scores will be concealed for the general practitioner. A computer randomized allocation sequence will be concealed until all general practices are assigned. Patients will be informed that they are allocated to the intervention arm or the control group by the research team through e-Boks.

### Blinding (masking)

All researchers working on quantitative analyses will be blinded to practice allocation. It is not possible to blind qualitative researchers as they analyze data they have collected themselves. Due to the nature of the intervention, it will not be possible to blind the participants or other members of the research team to allocation.

### Data collection before randomization

#### General practice data

Data collected about the general practices will include service number of the general practice (Danish: *ydernummer*), address, name(s) of general practitioners, number of patients registered at the practice, number of staff (including nursing staff and administrative staff).

#### Patient demographics and medical history

The following information will be recorded in REDCap by practice staff: CPR-number (Danish social security number), sex, age, ICPC-2 codes, and ICD-10 diagnostic codes. Reasons for non-eligibility will be recorded.

#### Quality of life measurements

For all recruited participants, the MMQ and EQ-5D-5L will be collected before allocation.

##### MMQ

The validated version of the MultiMorbidity Questionnaire (MMQ) encompasses two parts: MMQ1 and MMQ2. MMQ1 measures needs-based quality of life and MMQ2 measures self-perceived social inequity. MMQ1 encompasses 6 scales measuring: Physical ability (6 items); Worries (6 items); Limitations in everyday life (10 items); My social life (6 items), Self-Image (6 items); and Personal economy (3 items). The development of the items and the scales is based on the needs-based model for quality of life. MMQ2 encompasses different numbers of scales, with different numbers of items, that measure different constructs of self-perceived social inequity addressed towards five different types of contacts: general practitioners, the clinical staff at the general practice, other health professionals, the municipality employees, and finally, family, friends, and acquaintances.

##### EQ-5D-5L

The EQ-5D-5L is a standardized measure of health status developed by the EuroQol Group to provide a simple, generic measure of health for clinical and economic appraisal. The EQ-5D-5L descriptive system comprises the five dimensions: mobility, self-care, usual activities, pain/discomfort, and anxiety/depression. Each dimension has five response levels: no problems, slight problems, moderate problems, severe problems, and unable to/extreme problems. The respondent should indicate his/her health state by checking the box next to the most appropriate response level for each of the five dimensions [30].

### Data collection during the study period

#### Data from practices’ electronic medical records

From the practices’ electronic medical records, data will be recorded in REDCap by practice staff on patient characteristics, recruitment rates, response rates of consent forms, and attendance rates of patients in the intervention group. The SOFIA study’s trial research team provides support to practices and can monitor progress in each practice via REDCap in real-time.

#### Qualitative data on trial management, implementation, and program theory

Semi-structured interviews with general practitioners and staff will be conducted concerning the recruitment process, the educational course, the preparation and execution of the prolonged consultations, and at the end of the study. Observations will be performed of the information meetings with the practices, the educational course, and prolonged consultations in practice.

### End-of-study data collection

#### Mortality and morbidity data

Data on mortality and in- and outpatient hospital admissions during the study period in the participating patients will be collected from the National Patient Registries after completion of the pilot trial.

#### Response rates and quality of life measurements

For all recruited participants, the MMQ and the EQ-5D-5L will be collected at the end of the study period. Questionnaires will be either sent through REDCap to their e-Boks or by regular mail or email to participants in the intervention and control group at the end of the study period.

#### Medication

Data on the type of redeemed medication prescriptions from the 12 months before randomization and during the study period will be collected from the Danish National Health Service Prescription Database.

#### Biochemical and biometric data

Biochemical and biometric data available for the 12-month period before randomization, the study period, and up to 6 months after the study period will be collected concerning participants allocated to the intervention and control group. Electrocardiogram (EKG), serum hemoglobin A1C (HbA1c) serum HDL, LDL, and total cholesterol will be collected from a centralized clinical chemical laboratory database. Height, weight, and systolic and diastolic blood pressure values will be collected by the practice staff or the general practitioner from the electronic medical record and recorded in REDCap.

### Data management

The study will commence upon approval by the relevant Danish authorities and strictly adhere to Danish law governing medical research in humans and safeguarding of personal information of any kind. Data is stored and handled according to the General Data Protection Regulation. The study owner is responsible that all study activities comply with the Danish law on handling of personal data. The study owner is responsible that the identities of the participants are kept strictly confidential. To ensure these objectives, information that might allow identification of participants (e.g. name, CPR number, contact information) will also be encrypted and located on secure password-protected servers where all activity is subjected to transaction logging. All data that might allow identification of the participants will be stored for 25 years after termination of the study at which point it will be anonymized.

### Statistical methods

The quantitative parts of the primary objectives in this pilot study, e.g., the rate of recruitment of general practitioners and patients, retention of patients, and outcome data, will primarily contain descriptive statistics to assess the quality of these parameters: Mean (standard deviation) for continuously measured objectives, and raw count (%) for categorically measured objectives. For some objectives, the difference between the randomization groups is of interest, and this will be assessed statistically in linear or logistic regression models.

### Qualitative methods

Interviews will be recorded and transcribed, and notes will be taken systematically during observations. All qualitative data will be analyzed with qualitative methods ensuring a systematic and transparent approach and several researchers will cooperate and reach a consensus of the coding structure and analysis of the data. The software program NVivo will be used in the process of coding and analysis.

### Data monitoring

Due to the short duration of the trial and minimal health risks for the participating practices and patients, no formal data monitoring committee will be used. Interim analyses and auditing will not be performed.

### Harms

The study aims to improve the overall health and quality of life for a group of Danish citizens that are currently not receiving the indicated treatment for common conditions, and who experience the highest level of inequality and inequity in healthcare. The value of the study lies in its ability to identify and treat or support people with multiple conditions and to provide them with the appropriate clinical care to reduce the risk of premature death from untreated or undertreated somatic conditions.

A potentially harmful effect of our study is overdiagnosis. Since the study aims to identify and treat undiagnosed conditions, certain deviations, abnormalities, risk factors, or pathologies may be detected that were never going to cause harm. The limited sample size of the pilot trial prevents appropriate analyses of any potential harms and will therefore not be performed.

We expect that neither acceptance nor rejection from patients to participate in the study will affect the quality of clinical treatment by their general practitioner. The general practitioners that are allocated to the intervention arm will have received training in working with patients with SMI. Hence, it is anticipated that the care for the patients that do not wish to participate is better relative to the treatment they would have received otherwise.

### Modifications of protocol

Any modifications to the protocol that may affect the conduct of the study, the potential benefit of the patient, or patient safety, including changes of study objectives, study design, patient population, sample sizes, study procedures, or significant administrative aspects, will require a formal amendment to the protocol. Such amendments will be agreed upon by the steering committee and the primary funder and should be approved by the Ethics Committee before implementation.

Administrative changes to the protocol are minor corrections and/or clarifications that do not affect the way the study is to be conducted. These administrative changes will be agreed upon by the steering committee and the primary funder and will be documented in a memorandum.

### Consent

An initial enrolment visit at the general practices is offered to individuals responding positively to the initial invitation to participate. The initial enrolment visit is scheduled at the convenience of the respondent. The visit will be held in a standard consultation room safeguarded against interruptions, including telephones or other disturbing elements. The visit is held by a member of the clinical staff employed by the general practitioner. Information material about the trial and a copy of the consent form is sent to the potential participant before the appointment. At the initial enrolment visit, clinical staff provides key information about the trial and answers any questions. Instructions guiding the initial enrolment visit are made to ensure that adequate care is taken to present all essential background, practical details, and ethical considerations and that this information is understood by the patient.

### Confidentiality

All study-related information will be stored securely and electronically in REDCap, which complies with the demands set forth by the General Data Protection Regulation. This includes forms, lists, logbooks, appointment books, and any other listings that link participant ID numbers to other identifying information. All reports, data collection, process, and administrative forms will be identified by an identification number only to maintain participant confidentiality. All records that contain names or other personal identifiers, such as locator forms and informed consent forms, will be stored separately from study records identified by a code number on a secure server. All local databases will be secured with password-protected access systems. Participants’ study information will not be released outside of the study without the written permission of the participant.

### Access to data

The trial manager will grant access to quantitative data sets following individual assessment to ensure that members of the research team only have access to data that they need. Study data sets will be housed on REDCap and Secure Information Facility (SIF), a secure server hosted by the University of Copenhagen. To ensure confidentiality, data dispersed to research team members will be devoid of any identifying participant information. The qualitative data will be stored as detailed above in pseudonymized form and analyzed in anonymous form in the software NVivo by relevant researchers. The research group will guide the operational management of the trial, with responsibility for the overall supervision of the study.

### Dissemination policy

All results from the study (be it positive, negative, or inconclusive) will be published in peer-reviewed journals. The final list and order of authors follow the contribution from each researcher and follows the Vancouver rules and the guidelines from The Danish Committees on Scientific Dishonesty. In case the list and order of co-authors cannot be decided jointly by researchers, this decision will be made by the study owner. In case of disagreement on the validity, presentation, or interpretation of the results, each researcher is free to publish independently as outlined by the Danish Committees on Scientific Dishonesty.

### Trial status

The date of pilot trial protocol registration was 05/11/2020, and the registration number is NCT04618250. Recruitment of general practitioners started on November 6, 2020, and was approximately completed on March 15,2021.

## Discussion

The overarching goal of this cluster-randomized pilot trial is to determine the feasibility and acceptability of conducting a definitive randomized control trial to evaluate a coordinated co-produced care program in the general practice setting to reduce mortality and improve quality of life in patients with a severe mental illness. If delivery of the intervention proves feasible, a definitive trial to determine the effectiveness of the intervention can take place.

Our recruitment strategy might be biased towards patients who are already willing to receive and seek medical attention. Preferential recruitment of these patients will limit the generalizability of our findings and may reduce the observed efficacy of our intervention in a future definitive trial. Preferential selection of trial participants with favorable prognostic characteristics is widely acknowledged, yet typically ignored in trials [31]. In our pilot study, we, therefore, examine the risk of selective recruitment. If the risk of selective recruitment is present, we will re-evaluate and, if needed, adjust the recruitment strategy. Another limitation of the study design involves the randomization at the level of the general practice rather than at the individual patient. In the definitive trial, outcomes will be measured at the individual patient level, yet the intervention is targeted at the general practitioner and the practice staff. As staff and general practitioners will not be able to adapt care provision on an individual basis depending on allocation, randomization at the patient level will inevitably introduce contamination bias and lead to underestimation of the potential effectiveness of the intervention. However, randomization at a cluster level might challenge the comparability of the intervention and the control group [32]. Analysis of the definitive RCT will therefore require correction for potential imbalances in patient and practice characteristics.

The COVID-19 pandemic will likely influence the course and outcome of this pilot study. Until widespread immunization against COVID-19 is in place, resurgences of the pandemic will occasionally restrict access to primary health care for patients [33]. This will exacerbate the pre-existing barriers people with severe mental illness encounter when seeking medical attention, emphasizing the timeliness of research of feasibility, acceptability, and ultimately efficacy of interventions aimed to improve access to health care for this vulnerable patient group [34].

## Data Availability

Data sharing does not apply to this article as no datasets were generated or analyzed during the current study.

## Abbreviations

SMI: Severe mental illness
MMQ: MultiMorbidity Questionnaire
ICPC-2: International Classification of Primary Care version 2
